# Direct Blood Culturing of *Candida* spp. on Solid Medium by a Rapid Enrichment Method with Magnetic Beads Coated with Recombinant Human Mannan-Binding Lectin

**DOI:** 10.1128/JCM.00057-20

**Published:** 2020-03-25

**Authors:** Xiao-Li Chen, Hao Zheng, Wen-Ge Li, You-Hong Zhong, Xiao-Ping Chen, Jin-Xing Lu

**Affiliations:** a National Institute for Communicable Disease Control and Prevention, State Key Laboratory for Infectious Disease Prevention and Control, Collaborative Innovation Center for Diagnosis and Treatment of Infectious Disease, Chinese Center for Disease Control and Prevention, Beijing, China; b Yunnan Provincial Key Laboratory for Zoonosis Control and Prevention, Yunnan Institute for Endemic Disease Control and Prevention, Dali, China; Carter BloodCare & Baylor University Medical Center

**Keywords:** candidemia, mannan-binding lectin, direct blood culturing, magnetic enrichment

## Abstract

A rapid and accurate method to identify the species and antibiotic resistance of Candida spp. in blood is vital to increase the survival rates of patients with bloodstream infections. However, the extremely low levels of Candida spp. in blood make rapid diagnosis by standard blood culture difficult. In this study, we constructed a direct blood culturing method (i.e., the M1 method) by a rapid enrichment method with magnetic beads coated with a recombined human mannan-binding lectin (rhMBL; i.e., M1 protein), which demonstrated much higher *Candida* sp.-binding capacity than that of full-length MBL expressed *in vitro* (i.e., M2). With the M1 method, individual colonies were obtained before the standard blood culture method for each species of *Candida* spp. tested at <1 CFU/ml (an average of 29 h earlier). Additionally, the clinical sensitivity of the M1 method was 90.5% compared with that of the standard blood culture method when detecting frozen plasma from patients. More significantly, the turnaround time of the M1 method for blood culture could be reduced by approximately 37 to 43 h compared with that of the standard blood culture method in clinical sample identification.

## Abstract (precis)

A rapid and accurate method to identify the species and antibiotic resistance of Candida spp. in blood is vital to increase the survival rates of patients with bloodstream infections. However, the extremely low levels of Candida spp. in blood make rapid diagnosis by standard blood culture difficult. In this study, we constructed a direct blood culturing method (i.e., the M1 method) by a rapid enrichment method with magnetic beads coated with a recombined human mannan-binding lectin (rhMBL; i.

## INTRODUCTION

Candidemia is now the fourth leading cause of nosocomial bloodstream infections, with a mortality rate of approximately 50% (1–4). The current diagnostic standard comprises inoculation of the patient’s blood into special bottles with liquid medium and incubation in an automated blood culture (BC) instrument (5). Due to the low concentration of yeast (usually ≤1 CFU/ml) in the blood of most patients and the slow growth character of these pathogens, the turnaround time for the present BC method (i.e., liquid medium incubation) is frequently >48 h and can be up to 5 days (6). Once a growth signal is detected, the broth is further subcultured onto a solid medium to grow individual colonies for identification and antimicrobial susceptibility testing (7). This process results in a long time delay between sampling and obtaining a result, which will lead to delayed initiation of antifungal therapy and subsequent unfavorable outcomes (8). Though the advantages of direct culture on solid medium, which eliminates the incubation of blood samples in liquid medium, were anticipated a long time ago (9–11), few applicable methods are currently available.

Through its carbohydrate recognition domain (CRD), human mannan-binding lectin (MBL) can bind to mannose and *N*-acetylglucosamine residues found on various microbial surfaces, including *Candida* spp. (12). MBL can form various oligomeric structures, including dimers, trimers, tetramers, and hexamers, based on subunits comprising three identical 32-kDa peptide chains. Each peptide chain is composed of a CRD, a coiled-coil hydrophobic neck region (N region), a collagenous region, and a cysteine-rich N-terminal region (13). It was estimated that the interactions between separate CRDs and sugars are relatively weak. The avidity of the entire MBL protein for larger patterns of carbohydrates relies on the degree of oligomerization of the CRDs (i.e., the number and clustering of CRDs), while the higher oligomers have relatively low nanomolar *K_d_* (dissociation constant) values (14, 15). Tetrameric MBL was thought to have a higher pathogen-binding capacity than that of trimeric or dimeric MBL (16). MBL is present mainly as trimeric and tetrameric oligomers in serum (17).

Recently, a recombinant human MBL (rhMBL) was produced to capture pathogens from whole blood to enable their rapid detection (18). Here, we also constructed an rhMBL (i.e., M1) composed of 6 CRDs. Interestingly, M1 demonstrated a higher *Candida* sp.-binding capacity than did full-length MBL expressed *in vitro* (i.e., M2), most of which were tetramers with 12 CRDs. With the M1 method, direct blood culturing of *Candida* spp. on solid medium was successfully achieved much faster than with standard blood culture.

## MATERIALS AND METHODS

### M1 and M2 expression and purification.

The gene fragment of M1 was synthesized *in vitro* and comprised a 6×His tag, followed by methionine-alanine-lysine-threonine, a human IgG1 Fc fragment (residues C220 to G446), an Arg linker, and a human MBL2 fragment (residues 81 to 228 of the mature protein containing the neck and CRD region). To facilitate protein expression, cDNA from this construct was cloned downstream of the human interleukin 2 (IL-2) signal sequence in a PCDNA3.1 expression plasmid for amplification in TOP10 competent cells and in HEK293F cells for stable transfection, following the manufacturer’s protocols. M1 was purified from conditioned medium using a nickel-affinity chromatography column (GE Healthcare Life Sciences) on an ÄKTA avant system (GE Healthcare Life Sciences).

Similarly, the full sequence of human MBL2 with a 6×His tag was also synthesized *in vitro*. Then, this sequence was genetically engineered to develop an M2–Strep-tag II fusion protein, which consisted of the Strep II tag, 6×His tag, and full-length human MBL2. cDNA from this construct was cloned downstream of the human IL-2 signal sequence and expressed as described above. M2 was purified from conditioned medium through a nickel-affinity chromatography column (GE Healthcare Life Sciences) on an ÄKTA avant system (GE Healthcare Life Sciences).

Purified M1/M2 was dissolved in Tris-buffered saline (TBS). The concentrations of these proteins were analyzed with the bicinchoninic acid (BCA) method (BCA protein assay kit; Solarbio Biotech, Beijing, China).

### Preparation of *Candida* spp.

Accounting for more than 90% of the causative pathogens of candidemia (19), Candida albicans (ATCC 10231), Candida tropicalis (ATCC 750), Candida parapsilosis (ATCC 22019), Candida glabrata (ATCC 2001), and Candida krusei (ATCC 6258), as well as nine randomly selected clinical strains of each species, were used as targets to evaluate the binding capacity of rhMBL. Clinical strains were collected from two hospitals in Beijing, China, from 2014 to 2015. These strains were isolated from the blood of inpatients. The use of clinical strains was approved by the ethics committee of the National Institute of Communicable Disease Control and Prevention.

*Candida* spp. were cultured overnight at 25°C in yeast extract peptone dextrose (YPD) broth from Sabouraud glucose agar (SGA) plate stocks. All overnight cultures were inoculated at 1:20 to 1:30 dilutions in growth medium and cultured for another 2 to 3 h before the start of the experiment. Before use, fungal cells were washed four times with phosphate-buffered saline (PBS), and cell numbers were estimated using a hemocytometer.

Based on the CFU calculations performed in the preliminary experiments for each strain, various volumes (50 to 300 μl) of the yeast suspension were added to 1 ml of PBS and 10 ml of rabbit whole blood to produce PBS simulated samples (10 to 20 CFU/ml and 1 CFU/ml, respectively) or whole-blood simulated samples (≤1 CFU/ml). The actual yeast concentrations in these serial dilutions were confirmed by vital cell count on nutrient agar plates.

### Detection of M1/M2 binding to *Candida* spp.

Western blotting was used to qualitatively investigate the direct binding of M1/M2 to five species of *Candida* (20). Briefly, 1 mg/ml M1 or M2 (supplemented with 2 mM CaCl_2_) was incubated for 30 min with a suspension of *Candida* sp. (6 × 10^7^ cells) at room temperature with rotation. After harvesting and washing intensively four times with PBS, the bound M1/M2 was eluted from the pelleted cells by 7% sodium dodecyl sulfate (SDS). Each fraction was subjected to analysis by 12% SDS-PAGE gels under reducing conditions, followed by Western blotting using an anti-MBL antibody (ab189856; Abcam, Cambridge, UK). Fungal cells incubated with PBS alone were used as a control.

The quantitative binding of M1/M2 to *Candida* spp. was determined using a flow cytometric procedure with some modifications (see the Supplemental Methods).

### M1/M2 bead preparation and capture efficiencies with PBS simulated samples.

Using an Fc-based or biotin-based purification technique, M1 or M2 was efficiently immobilized on paramagnetic beads coated with protein A or streptavidin, respectively. Briefly, protein A-coated magnetic beads (1.5 μm; PuriMag Biotech, Hangzhou, China) or streptavidin-coated magnetic beads (1.5 μm; PuriMag Biotech) were incubated with M1 or M2, respectively, for 30 min at room temperature, and the remaining unbound sites on the beads were blocked with 1% bovine serum albumin (BSA) at 4°C for 1 h. Thus, the N terminus of M1/M2 attached to the bead surface, and the CRD faced outward. The M1/M2 beads (10 mg beads ml^−1^) were resuspended in PBS and stored at 4°C for less than 4 h before use.

To compare the capture efficiency of M1 beads with that of M2 beads, PBS simulated samples (10 to 20 CFU/ml *Candida*) were used to determine the recovered colonies with these two beads. Furthermore, PBS simulated samples (1 CFU/ml *Candida*), which were more relevant to a clinical setting, were also evaluated with the M1 bead capture method (Supplemental Methods). Scanning microscopy was applied to demonstrate the interaction between M1/M2 beads and C. glabrata as an example (Supplemental Methods).

### M1 bead-based direct culture method with rabbit blood simulated samples.

Thirty certified *Candida*-free specific-pathogen-free (SPF) male and female New Zealand White rabbits (2.5 to 3.5 kg) were selected and housed in individual cages and acclimated to a 12-h/12-h light:dark cycle in a temperature- and humidity-controlled SPF environment. The environment was controlled at 18 to 22°C. Rabbits were maintained according to the guidelines of the Animal Research Committee in Chinese Center for Diseases Control and Prevention, and food and water were provided *ad libitum*.

Blood (5 ml from each animal) was collected from the vein in the ear of the rabbits with plastic heparin lithium spray-coated lavender BD Vacutainers. The tubes were gently inverted, end over end, 6 times at the time of blood collection.

In our pretest, 1 mg of protein A beads was saturated with 232 μg of M1. Then, to construct M1 beads, 2 mg of protein A beads was immobilized with 464 μg of M1 protein. An equal volume of binding buffer (1× PBS with 4 mM CaCl_2_ [pH 7.4]) was added to 10 ml of blood simulated sample (*Candida* sp. concentration, ≤1 CFU/ml). Next, 2 mg of M1 beads was added to the sample. To eliminate the influence of blood IgG on the protein A beads, bare beads blocked with 1% BSA were used as negative beads. Meanwhile, the same volume of the yeast suspension as the volume added to the simulated blood samples was also plated on YPD agar plates in triplicate as the control. The remaining procedures were as described above.

Standard blood cultures of 10 ml of blood simulated samples containing ≤1 CFU/ml of various *Candida* species were also conducted in a BacT/Alert3D60 system (bioMérieux, France) with the BacT/Alert SA bottle, according to the manufacturer’s instructions. The time to obtain individual colonies (TTIC) was defined as the time from when the bottles were inoculated in the Bactec unit or M1 beads were added to the blood samples until the same size individual colonies formed on the YPD agar plates. The colony size, which was suitable for matrix-assisted laser desorption ionization–time of flight mass spectrometry (MALDI-TOF MS) identification, was determined simultaneously by two experienced technicians. After individual colonies formed, typing and definite species identification with MALDI-TOF MS were performed on a Microflex LT mass spectrometer (Bruker Daltonics, USA) with the BioTyper software v2.0, using default parameter settings.

### Evaluation of the M1 method with clinical samples.

**FIG 1:**
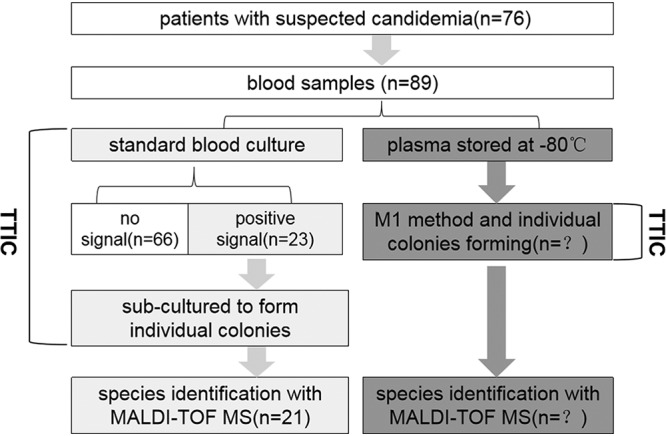
Overview of the evaluation of the M1 method with clinical samples.

This study was approved by the ethics committee of the National Institute of Communicable Disease Control and Prevention and was conducted as a retrospective anonymous case-control study to assess the performance of the M1 method, with no impact on patient management. A summary of the process used to evaluate the M1 method with clinical samples is shown in Fig. 1. A total of 87 frozen (–80°C) plasma samples, which were collected from 76 patients at hospitals in Beijing over an 18-month period from February 2017 to September 2018, were tested by the M1 method. The patients were a combination of cardiopulmonary disorder patients (*n* = 33), hematology patients (*n* = 22), neurological disorder patients (*n* = 14), and gastrointestinal disorder patients (*n* = 7). These frozen plasma samples were collected on the same day that blood was drawn for standard blood culture. Standard blood cultures were conducted in the BacT/Alert3D60 system (bioMérieux, France) with the BacT/Alert SA bottle, and a recommended blood volume of 8 to 10 ml for each sample was added to the bottle. The TTIC, which was retrospectively collected, was defined as the time from when bottles were inoculated in the Bactec unit until individual colonies suitable for MALDI-TOF MS analyses formed on the YPD agar plates.

The M1 method was performed according to the method with simulated rabbit blood samples. Briefly, after thawing at room temperature, 3 to 5 ml of plasma from each sample was added to an equal volume of binding buffer and M1 beads. The remaining procedures were the same as those described above. The TTIC was defined as the time from when M1 beads were added to the blood samples until the same size individual colonies formed on the YPD agar plates. Determination of the colony size suitable for MALDI-TOF MS identification was performed simultaneously by two experienced technicians. After individual colonies formed, typing and definite species identification with MALDI-TOF MS were performed as described above.

### Data analyses.

Data were analyzed by Student’s *t* tests. The differences were not considered to be significant if the *P* values were greater than 0.05. A 2 by 2 contingency table was also used to calculate the sensitivity, specificity, and positive and negative predictive values (21).

### Data availability.

The cDNAs of M1 and full sequence of human MBL2 are available at GenBank (accession numbers MN399862 and MN399863).

## RESULTS

### The M1 protein formed polymers with 6 CRDs, while most of the M2 protein formed tetramers.

**FIG 2:**
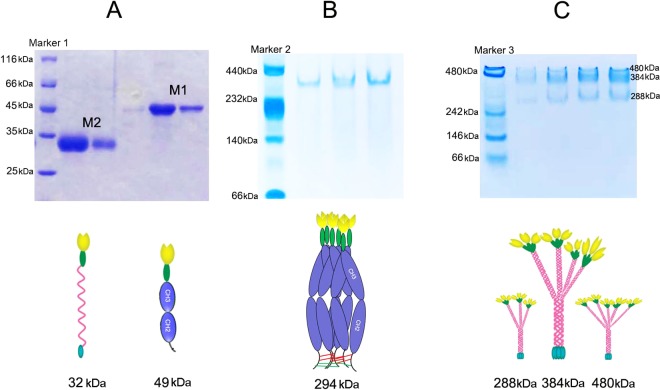
(A to C) Purified M1 and M2 were analyzed by SDS-PAGE (A) and native PAGE of M1 (B) and M2 (C). The corresponding molecular pattern diagrams are shown under each PAGE figure. The M2 monomer was 32 kDa, the M1 monomer was 49 kDa, and the M1 polymer was 294 kDa, with 6 monomers. Red lines at the C terminus indicate two disulfide bonds in the hinge region, while red lines indicate disulfide bonds formed by residue C220; most M2 formed tetramers (middle thick band), and small portions formed trimers (lower light band) or pentamers (higher light band).

By expressing M1/M2 from HEK293F cell lines and using a single-step, 6×His-based purification technique, large amounts of single-band-quality M1 (0.15 to 1.0 mg/ml) or M2 (0.2 to 0.5 mg/ml) were produced. Figure 2A shows the monomers of M1 (49 kDa) and M2 (32 kDa) by SDS-PAGE. We further investigated their state of polymerization with native PAGE. As shown in Fig. 2B and C, the molecular weight of M1 was approximately 294 kDa, which is equal to 6 monomers, while most M2 showed a molecular weight of 384 kDa (tetramer); a small portion of M2 was 288 kDa (trimer) or 480 kDa (pentamer).

### M1 had a much higher *Candida* sp.-binding efficiency than did M2.

**FIG 3:**
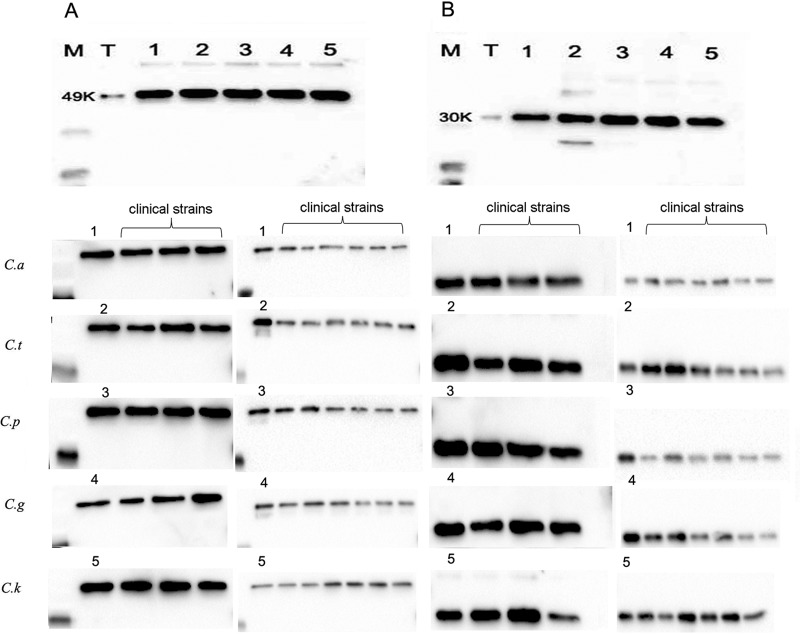
Direct binding of purified rhMBL to *Candida* spp. as analyzed by Western blotting. (A and B) Binding of M1 (A) and M2 (B) to 5 species of *Candida* in the presence of 5 mM CaCl_2_. T, M1 or M2; 1, C. albicans (*C.a*; ATCC 10231) binding protein; 2, C. tropicalis (*C.t*; ATCC 750) binding protein; 3, C. parapsilosis (*C.p*; ATCC 22019) binding protein; 4, C. glabrata (*C.g*; ATCC 2001) binding protein; 5, C. krusei (*C.k*; ATCC 6258) binding protein; clinical strains, various clinical strains binding protein. M, marker.

First, Western blotting was performed to study the direct binding of M1/M2 with 5 species of *Candida*. As shown in Fig. 3, in addition to the five species of the *Candida* ATCC strains that bound to M1 (Fig. 3A) and M2 (Fig. 3B), all nine randomly selected clinical strains of each species of *Candida* also bound to M1 and M2. Next, flow cytometric analyses were applied to quantitatively investigate the binding. A representative flow cytometry overlay plot for 10 μg/ml M1/M2 is shown in Fig. S1A in the supplemental material. The results from three independent experiments are shown in Fig. S1B. For all 5 species of *Candida*, M1 had a higher binding capacity than did M2. The addition of EDTA demonstrated a marked decrease in both M1 and M2 binding.

PBS simulated samples (10 to 20 CFU *Candida*/ml) were used to compare the capture efficiency of M1 beads with that of M2 beads. As shown in Fig. S2A, M1 beads (6 μg of protein plus 10 μl of protein A beads) could achieve capture efficiencies of approximately 58% for C. parapsilosis, 80% for C. albicans, 90% for C. tropicalis and C. krusei, and 100% for C. glabrata. The capture efficiency of the M2 beads (6 μg of protein plus 10 μl of streptavidin beads) was significantly lower than that of the M1 beads. Further assays indicated that M1 beads could capture all five species of *Candida* from 6/6 PBS simulated samples (*Candida* spp., 1 CFU/ml) (Fig. S2B). Scanning electron microscopy also showed the direct binding of M1 or M2 to C. glabrata as an example of direct binding (Fig. S3). The capture performance was consistent across these batches of M1 beads.

### Direct blood culturing of *Candida* spp. on solid medium was achieved by a rapid enrichment method with M1 magnetic beads.

**FIG 4:**
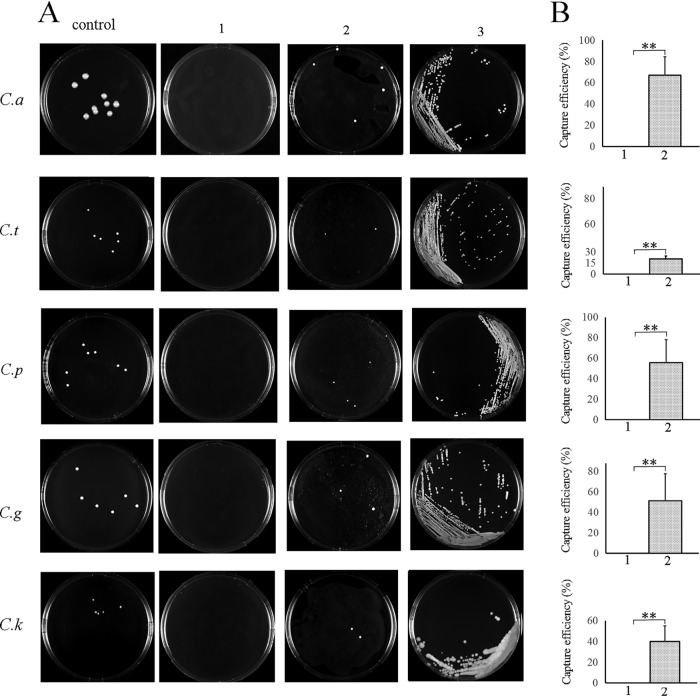
(A) Representative images of colonies recovered with M1 beads in 10 ml of blood with 1 CFU/ml *Candida* sp. (B) Capture efficiency (%) of M1 beads in 10 ml of blood with 1 CFU/ml C. albicans (ATCC 10231), C. tropicalis (ATCC 750), C. parapsilosis (ATCC 22019), C. glabrata (ATCC 2001), and C. krusei (ATCC 6258). Capture efficiency was assessed by the ratio of the recovered colony numbers with magnetic beads to the mean of three colony numbers recovered from the equal volume of serial dilutions added to simulated blood samples. Data are the mean ± standard deviation (SD) values of the results from assays performed in 5 to 6 repetitions. 1, captured with 2 mg of negative beads; 2, captured by 2 mg of protein A beads with 464 μg of M1 protein; 3, individual colonies formed with the standard blood culture method. **, *P* < 0.01.

Because M1 showed much higher *Candida* sp.-binding capacity than did M2 in the above-described experiments, M1 was used in the following study. As shown in Fig. 4A, 2 mg of M1 beads (with 464 μg of M1 protein) could capture 1 to 9 colonies from 10 ml of whole blood with ≤1 CFU/ml *Candida* sp. The M1 beads had capture efficiencies of approximately 67% for C. albicans, 50% for C. glabrata and C. parapsilosis, 40% for C. krusei, and approximately 20% for C. tropicalis, which were significantly higher than the capture efficiencies of the negative beads (Fig. 4B). Overall, individual colonies with the M1 method were achieved before the standard blood culture method for each species of *Candida* at ≤1 CFU/ml concentrations tested (an average of 29 h earlier) (Table 1).

**TABLE 1:** Comparison of TTIC using the M1 method with the standard blood culture method^*a*^

Pathogen	Total no. of pathogens in 10 ml whole blood (≤1 CFU/ml)	Data for M1 method^*b*^		Standard blood culture TTIC (h) (positivity signal + individual colony forming)
		Capture efficiency (mean ± SD) (%)	TTIC (h)	
C. albicans	9–10	67.6 ± 17.7	49.5	82.5 (33.0 + 49.5)
C. glabrata	5–10	51.5 ± 26.4	27.5	54.5 (27.0 + 27.5)
C. parapsilosis	5–10	55.6 ± 22.3	47.5	78.6 (31.1 + 47.5)
C. tropicalis	5–9	20.9 ± 3.7	27.6	50.2 (22.6 + 27.6)
C. krusei	2–8	40.0 ± 14.9	18.5	50.7 (32.2 + 18.5)

a TTIC, the time to obtain individual colonies.

b The M1 method detected all 6 of 6 samples.

### Clinical evaluation demonstrated that the M1 method was more rapid than the standard blood culture method, while the efficiencies were similar.

A total of 89 plasma samples were detected with the M1 method. Among these samples, individual colonies (1 to 3 colonies per sample) were successfully recovered from 19 plasma samples with the M1 method. Species identification was conducted with MALDI-TOF MS, and the results were consistent with those obtained with the standard blood culture method plus MALDI-TOF MS (Table S1). Only 2 plasma samples were not detected with the M1 method. Compared with that of the standard blood culture method, the clinical sensitivity of the M1 method was 90.5%, the specificity was 100%, the positive predictive value (PPV) was 100%, and the negative predictive value was 97.1% (Table 2). The TTIC of the M1 method was significantly less than that of the standard blood culture method, except for C. tropicalis, whose sample number was too small for statistical analysis (Table 3). Overall, compared with the standard blood culture method, the M1 method saved approximately 37 to 43 h.

**TABLE 2:** M1 method compared to the standard culture method^*a*^

M1 method result	Standard blood culture method match (no. [%]) by result	
	+	−
+	19 (21.3)	0 (0)
−	2 (2.3)	68 (76.4)

a A total of 89 clinical specimens were included in the study. −, negative; +, positive. Standard blood culture was considered the gold standard. The analytical sensitivity was 90.5%, positive predictive value (PPV) was 100%, analytical specificity was 100%, and the negative predictive value (NPV) was 97.1%.

**TABLE 3:** Comparison of TTIC using the M1 method with the standard blood culture method^*a*^

Pathogen (*n*)	TTIC (mean ± SD) (h) for:		*P* value
	Standard blood culture	M1 method	
C. albicans (10)	86.0 ± 7.3	49.1 ± 0.9	<0.01
C. glabrata (5)	62.9 ± 5.7	27.8 ± 1.5	<0.01
C. parapsilosis (3)	91.3 ± 2.5	48.0 ± 3.6	<0.01
C. tropicalis (1)	65.0	23.0	NA^*b*^

a TTIC, the time to obtain individual colonies.

b NA, not applicable.

## DISCUSSION

The Fc of IgG1 is a very attractive scaffold for the design of recombined proteins with significant therapeutic potential. Fc-fused monomers can easily be created by fusing a monomeric protein to the N or C terminus of one Fc chain (22). Most Fc fusions are expressed as homodimers due to two disulfide bonds in the hinge region (23). Interestingly, M1 was demonstrated to be a polymer with 3 homodimers (i.e., 6 CRDs) by native PAGE. We deduced that noncovalent Fc-Fc interactions (large, tightly packed interfaces between identical CH3 domains) might contribute to the formation of M1 polymers (24, 25). Moreover, the IgG1 Fc N terminus of M1 in this study was not the same as those created by Kang et al. (26). In other studies, the 5th cysteine (C220) from the N terminus of IgG1, which forms disulfide bonds with another cysteine in the light chain, was either mutated or omitted (26–28). In our M1 design, residue C220 was preserved, which left 2 free cysteine residues in the N terminus of each homodimer. These free residues were very unstable when exposed to solvent solution (29) and easily formed disulfide bonds with the free cysteine residues in another homodimer, which might further contribute to the formation of polymers.

Obviously, this finding is very significant because higher-order structures of MBL have better binding capacity than do lower-order structures (14). Interestingly, as demonstrated by cytoflow analysis and *Candida* capture experiments in PBS simulated samples, M1 had a much higher *Candida* sp.-binding capacity than did M2, which was closer to that of native MBL. As revealed by native PAGE, most of the M2 formed tetramers. We supposed that the affinity depends not only on the degree of CRD oligomerization but also on the spatial structure formed by clustering of CRDs because a proper array of CRDs would more easily interact with the sugar patterns on the microbial surface (14). More studies should be performed to reveal the mechanism.

With M1-coated magnetic beads, we established a workflow that makes it possible to achieve direct blood culturing of *Candida* on solid medium from as low as ≤1 CFU/ml of *Candida* sp. in 10 ml of whole blood, which is very close to the situation in the bloodstream in a majority of patients (30). Significantly, the M1 method took only 18 to 49 h to obtain individual colonies on solid medium, which reduced the time by approximately 29 h compared with that of standard BC. At present, magnetic capture techniques have shown promising results, with the advantage of eliminating all other materials except for pathogens (26, 31, 32), and they are more effective than other enrichment methods, such as hollow disk separation and lysis-centrifugation methods (33, 34). Moreover, MBL has a broader spectrum of pathogen-capture ability than do other molecules such as antibodies and ApoH (35).

Finally, the M1 method showed clinical application value because its analytical sensitivity reached 90.5% compared with that of the standard blood culture method when detecting frozen plasma from patients. It is reported that for standard clinical BC, it usually takes >48 h to obtain a sufficient concentration (10^5^ to 10^8^/ml) of *Candida* spp. before being subcultured onto agar plates to obtain individual colonies (33), which was consistent with our retrospectively collected data (53 to 96 h, including 24 to 40 h needed for individual colony formation). Our results indicated that the M1 method, which eliminated the incubation of blood samples in liquid medium, saved approximately 35 to 43 h compared to the time required for the standard blood culture method. Though MALDI-TOF MS identification directly with positive blood culture has been used more widely in recent years, it is still difficult to analyze positive blood culture with polymicrobial infection (36). Therefore, in this retrospective study, the standard blood culture method still included the time for individual colony formation. It can be said optimistically that M1 protein, whose MBL component would bind different pathogens, is a promising tool to detect polymicrobial infection in blood. Interestingly, despite having similar *Candida* concentrations (≤1 CFU/ml), the TTIC of blood simulated samples was shorter than that of clinical samples for the standard blood culture method. We suspected that many factors, such as antibiotics in patient samples, influenced the results. However, the TTIC of blood simulated samples was the same as that of clinical samples for the M1 method, which indicated that the M1 method might eliminate the negative effects of antibiotics. Finally, we must acknowledge that more clinical samples, especially whole-blood samples with single or multiple pathogens, should be tested with the M1 method and compared with standard BC.

## Supplementary Material

Supplemental file 1
