# Supplementary material for: Direct Blood Culturing of Candida spp. on Solid Medium by a Rapid Enrichment Method with Magnetic Beads Coated with Recombinant Human Mannan-Binding Lectin
Source: J Clin Microbiol. 2020 Mar 25;58(4):e00057-20. doi: 10.1128/JCM.00057-20 (PMC7098737; doi:10.1128/JCM.00057-20)
Supplement: Supplemental file 1 [file JCM.00057-20-s0001.pdf]

Supplementary Methods:

Flow cytometry was used to analyze the binding of M1/M2 to *Candida*[1].

First, 100 µl of Candida suspensions (~1×10^6 CFU/ml) were centrifuged at 3000×g for 1 min and washed with 500 µl of veronal-buffered saline (VBS) supplemented with 5 mM $CaCl_2$ and 5 mM $MgCl_2$ (VBS^{2+}) (X-Y Biotechnology, Hangzhou, China). The pellet was resuspended in VBS^{2+} buffer with or without 10 µg/ml purified M1/M2. The suspensions were incubated at 37°C for 30 min. The Candida suspensions were then centrifuged, and the cell pellet was washed and resuspended in 100 µl of VBS^{2+} containing 5 µg/ml Alexa Fluor® 488-anti-6xHis antibodies (R&D, IC0501G, MN, USA). This mixture was incubated in the dark at 37°C for 20 min; then, the mixture was centrifuged, and the Candida cell pellet was washed. The samples were resuspended in 200 µl of VBS^{2+} and fixed by the addition of 200 µl of 2% (wt/vol) paraformaldehyde in PBS. The samples were kept at 4°C in the dark until analysis. Flow cytometric analyses were performed on a BD FACSCalibur flow cytometer (Beckton Dickinson), which was equipped with a 633 nm red laser and a 488 nm blue laser. Light transmission data from the Alexa Fluor® 488-illuminated MBL-Candida complexes were collected by a forward scatter (FSC) detector, side scatter (SSC) detector, and Alexa Fluor® 488 fluorescence detector. MBL binding was expressed as Alexa Fluor® 488 median fluorescence intensity (MFI).

To evaluate whether the binding observed was mediated by C-type lectin

interactions, inhibition experiments using the calcium-chelating agent EDTA were simultaneously performed. EDTA (10 mM) was added to the M1/M2 solution 10 min prior to the addition of M1/M2 to 5 different Candida species. MBL binding was expressed as Alexa Fluor® 488 MFI as in the above experiment. All binding experiments were repeated three times.

M1/M2 bead enrichment and culturing protocol with PBS-simulated samples.

Each experiment included 5-6 sample replicates (interassay), and beads were prepared freshly before each assay (i.e., each experiment contained 5-6 batches of beads). To compare the capture efficiency of the M1 beads to that of the M2 beads (positive beads), 1 ml of PBS containing different Candida species (10-20 CFU/ml) was prepared as a PBS-simulated sample. Equal amounts (6 μg) of M1/M2 were incubated and immobilized on 100 μg of the corresponding beads as described above. The negative beads (protein A-beads or streptavidin-beads without M1 or M2) demonstrated some degree of false positive results in PBS with 5 mM $CaCl_2$ in the pre-experiments, while both positive beads and negative beads had satisfactory results when incubated with 2 mM $CaCl_2$. Then, 2 mM $CaCl_2$ was used for the following capturing experiments. The M1/M2 beads were added to the PBS (containing 2 mM $CaCl_2$)-simulated sample. The sample was then mixed by inversion on a rotor for 30 min at room temperature. After incubation, the sample tube was magnetized for 5 min on a 15 ml magnetic rack until all magnetic beads were

concentrated on the side of the tube. The supernatant was discarded without disturbing the beads. The microbe-bound beads were then washed with 1 ml of PBS (pH 7.4). Next, the tube was placed on a magnetic rack for 5 min, after which the PBS was removed and discarded. The PBS wash was repeated once more. The microbe-bound, washed M1/M2 beads were then resuspended in 200 µl of PBS and grown on YPD agar plates to determine the number of Candida recovered from the PBS-simulated sample. Meanwhile, 1 ml of simulated PBS sample was also plated on YPD agar plates in triplicate as a control. The capture efficiency was determined as the ratio of the number of colonies recovered by magnetic beads to the mean average of three PBS-simulated samples. To prevent any incidental contamination from the environment, the entire process from microbial spiking until plating was carried out inside a microbiological laminar flow hood.

To evaluate whether 1 CFU/ml *Candida* spp. in 1 ml of PBS could be captured by 6 µg of M1, and the enrichment procedure was performed as described above.

Scanning electron microscopy.

C. glabrata were diluted to 10^6 CFU/ml in PBS (containing 5 mM $CaCl_2$) and mixed with 100 µg of negative beads or beads coated with 6 µg of M1 or 6 µg of M2. The samples were incubated on a rotor, end over end, for 30 min at room temperature. Then, the beads were separated with a magnetic separator rack and resuspended in 100 µl of 2.5% glutaraldehyde in 0.1 M cacodylate

buffer (pH 7.2). The samples were adhered to glass cover slips for at least 20 min at room temperature, dehydrated in a graded acetone series, and critical point-dried. After coating with a 20 nm thick gold layer in a sputtering device, the samples were examined with a JEOL scanning electron microscope (SEM), model JSM 6010PLUS-LA.

Supplementary Table 1. Colony numbers recovered by the M1 method and species identification by MALDI-TOF MS compared with those of the standard blood culture method plus MALDI-TOF MS.

	M1 method		Standard blood culture method
	Recovered colony numbers	Species identified by MALDI-TOF MS	Species identified by MALDI-TOF MS
Sample 1	1	*C. albicans*	*C. albicans*
Sample 2	1	*C. albicans*	*C. albicans*
Sample 3	1	*C. albicans*	*C. albicans*
Sample 4	2	*C. albicans*	*C. albicans*
Sample 5	1	*C. albicans*	*C. albicans*
Sample 6	2	*C. albicans*	*C. albicans*
Sample 7	1	*C. albicans*	*C. albicans*
Sample 8	1	*C. albicans*	*C. albicans*
Sample 9	2	*C. albicans*	*C. albicans*
Sample 10	3	*C. albicans*	*C. albicans*
Sample 11	1	*C. glabrata*	*C. glabrata*

Sample 12	2	*C. glabrata*	*C. glabrata*
Sample 13	1	*C. glabrata*	*C. glabrata*
Sample 14	1	*C. glabrata*	*C. glabrata*
Sample 15	2	*C. glabrata*	*C. glabrata*
Sample 16	2	*C. parapsilosis*	*C. parapsilosis*
Sample 17	1	*C. parapsilosis*	*C. parapsilosis*
Sample 18	1	*C. parapsilosis*	*C. parapsilosis*
Sample 19	1	*C. tropicalis*	*C. tropicalis*
Sample 20	ND	ND	*C. glabrata*
Sample 21	ND	ND	*C. albicans*

ND, not detected.

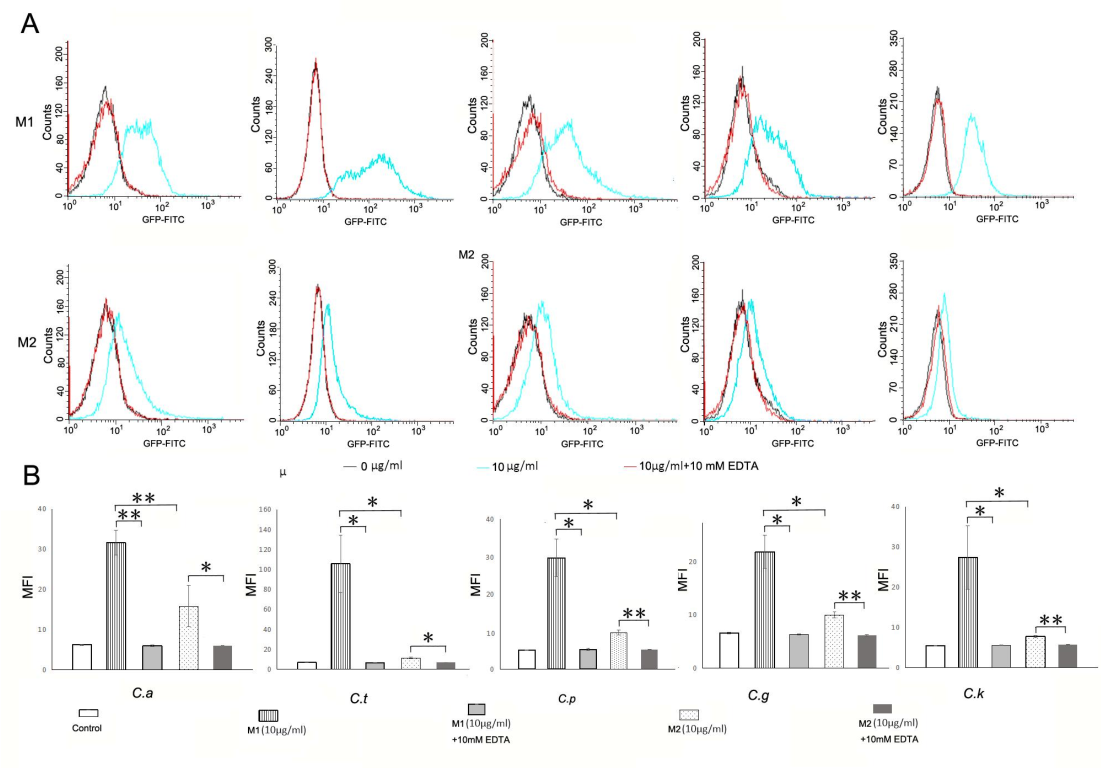

Sup Fig 1. Binding specificity of rhMBL to 5 species of Candida. (A) Representative flow cytometric overlay showing the binding of M1 or M2 to 5

species of Candida, *C. albicans* (ATCC 10231), *C. tropicalis* (ATCC 750), *C. parapsilosis* (ATCC 22019), *C. glabrata* (ATCC 2001), and *C. krusei* (ATCC 6258). The black lines represent the negative control without rhMBL, the blue lines represent samples with 10 μg/ml rhMBL alone, and the red lines represent samples with 10 μg/ml rhMBL and 10 mM EDTA, which were added prior to the rhMBL opsonization of fungal strains. (B) The average rhMBL-binding results for *C. albicans* (ATCC 10231), *C. tropicalis* (ATCC 750), *C. parapsilosis* (ATCC 22019), *C. glabrata* (ATCC 2001), and *C. krusei* (ATCC 6258) based on median fluorescence intensity (MFI). The results are the average of three separate experiments and are shown as the mean±SEM. *, P<0.5; **, P<0.01.

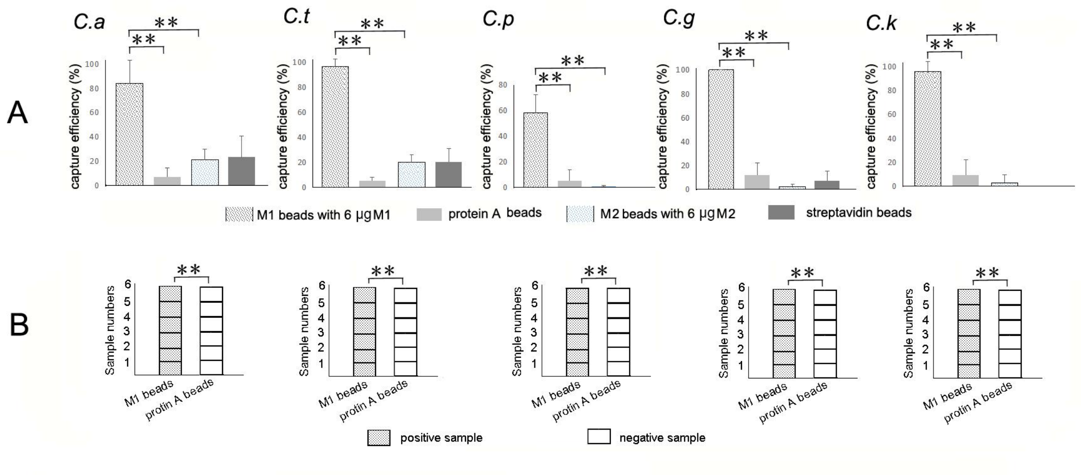

Sup Fig 2. A. Capture efficiency (%) of M1/M2 beads with equal quantities (6 μg) of M1/M2 protein in 1 ml of PBS with low concentrations (10-20 CFU/ml) of Candida. Capture efficiency was assessed by the ratio of the recovered colony numbers with magnetic beads to the mean average of the three PBS-simulated samples. Data are the mean±SD of assays performed in 5-6

repetitions at different times; **B**. Positive sample numbers of 1 ml of PBS with 1 CFU of Candida detected by M1 beads with 6 μg of M1 protein. Data are the summary of assays in 6 repetitions and analyzed by chi-square test. *C.a, C. albicans* (ATCC 10231); *C.t*, *C. tropicalis* (ATCC 750); *C.p*, *C. parapsilosis* (ATCC 22019); *C.g*, *C. glabrata* (ATCC 2001); *C.k*, *C. krusei* (ATCC 6258). **, $P<0.01$.

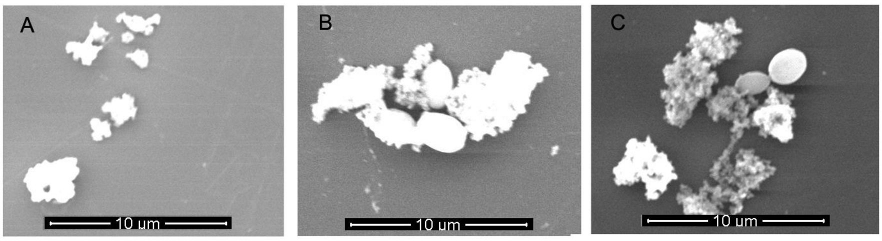

Sup Fig 3. Interaction between M1/M2 beads and *C. glabrata* as shown by scanning electron microscopy. Fungi (10^6 CFU) were incubated in 1 ml of PBS buffer with M1 or M2 magnetic beads (100 μg). A=Negative control; B=M1 bead-captured sample; C=M2 bead-captured sample. Scale bar=10 μm.

Reference:

1. Ulrich-Lynge SL, Dalgaard TS, Norup LR, Song X, Sørensen P, Juul-Madsen HR. 2015. Chicken mannose-binding lectin function in relation to antibacterial activity towards Salmonella enterica. Immunobiology 220:555-563.
